# Social inequalities in health behaviors among Brazilian adults: National Health Survey, 2013

**DOI:** 10.1186/s12939-016-0439-0

**Published:** 2016-11-17

**Authors:** Marilisa Berti de Azevedo Barros, Margareth Guimarães Lima, Lhais de Paula Barbosa Medina, Celia Landman Szwarcwald, Deborah Carvalho Malta

**Affiliations:** 1 State University of Campinas – UNICAMP, Campinas, Brazil; 2 Foundation Institute Oswaldo Cruz – FIOCRUZ, Rio de Janeiro, Brazil; 3 Federal University of Minas Gerais – UFMG, Belo Horizonte, Brazil; 4 Department of Public Health – UNICAMP - Tessália Vieira de Camargo, 126 - Cidade Universitária Zeferino Vaz, 13083-887 Campinas, SP Brazil

**Keywords:** Health behavior, Social inequalities, Smoking, Alcohol, Leisure-time physical activity, Diet

## Abstract

**Background:**

Considering the high socioeconomic inequalities prevailing in Brazil and lifestyle as a strong determinant of morbidity and premature mortality, our purpose was to evaluate the degree of socioeconomic disparities in the prevalence of health behaviors among Brazilian adult population using data from the 2013 Brazilian National Health Survey.

**Method:**

Based on a sample of 49,025 individuals aged 20 to 59 years, we estimated the prevalence of several health behaviors and a score of unhealthy behaviors according to gender, education, race/color and possession of private health insurance. The prevalence ratios adjusted by age and gender were estimated by means of multiple Poisson regression and the analyses took into account the sampling design.

**Results:**

Significant social inequalities were identified in the Brazilian adults. Higher prevalence of current smoking, leisure-time physical inactivity, sedentary lifestyle, whole milk consumption and low ingestion of greens, vegetables, and fruits were observed among the less educated, in the non-white population, and among those without private health insurance. Higher prevalence of heavy episodic drinking was found in the non-white population, but no difference in the consumption of fatty meat was found according to skin color. Score of unhealthy behavior higher than 6 was more frequent in lower educational strata (*PR* = 3.74) in the non-white population (*PR* = 1.39) and among those without private health insurance (*PR* = 1.78). Compared to women, men had higher prevalence rates of smoking, hazardous alcohol consumption, and fatty meat consumption and lower consumption of greens, vegetables and fruits.

**Conclusion:**

The results of the study emphasize the importance of monitoring social inequalities in health as part of national health policies and the urgent need to prioritize actions to promote healthy behaviors, especially among the most socially vulnerable segments of society.

## Background

The surveillance of social inequalities in health is an essential task in all countries, but particularly important in countries with high rates of income inequality [1]. Social determinants shape the health profile of the population, the adoption of health-related behaviors, and the organization and performance of healthcare systems. The literature on the social determinants of health has grown substantially in the last decades [2], highlighting the central role of health inequalities based on socioeconomic status, social class, gender, race/ethnicity and geography, among others [3, 4]. Current evidence reveals these inequalities are substantial across different population groups in most health-related behaviors and outcomes [5–9].

The Commission on the Social Determinants of Health (CSDH) has emphasized the need to incorporate the issue of health inequality into governments' political agendas [6, 7, 10]. The literature suggests that the magnitude of social inequalities varies with the type of health problem evaluated and with the socio-demographic subgroups undergoing comparison. Furthermore, multiple cultural and context variables influence, shape and interact with the effect of the socio-economic gradient on health [11]. In Brazil, analysis of health differences between segments with and without private health insurance provides an opportunity to assess the performance of the national health system - the *Sistema Único de Saúde* (SUS) - and to investigate the potential effect of national health policies on reducing health inequalities.

Today, a substantial part of the social differences in morbidity and mortality results from uneven patterns of health-related behaviors [12]. National and international literatures record the extent of social inequalities in the main risk factors for chronic diseases: smoking, physical inactivity/sedentary lifestyle, poor diet and harmful use of alcohol [9, 13–16]. In Australia, Ding et al. [14] observed reductions in the prevalence of harmful behaviors, from 2002 to 2012, coupled with an increase in inequalities across socioeconomic groups. In Spain, Bartoll et al. [15] observed improvements in the prevalence of healthy behaviors between 2001 and 2012, with varying degrees depending on the specific behavior, but confirmed that improvements were unequal across social groups.

Social inequalities in the prevalence of health-related behaviors may increase due to different temporal trends among social groups. Studies have shown that even when prevalence rates of harmful behaviors decline over time, disparities among social groups may increase [14, 17]. For example, despite Brazilian achievements in smoking cessation, the strongest decline in smoking prevalence occurred among individuals from higher socioeconomic status, which increased relative social disparities in tobacco-related diseases and deaths [18]. This may result from the fact that higher levels of nicotine addiction have been found among the most excluded and marginalized social groups [13].

The impact of unhealthy behavior on diseases and mortality rates is widely recognized [19, 20]. According to the World Health Organization (WHO) in 2012, about 6 % of all deaths globally were attributable to alcohol consumption and 12 % of deaths among men and 7 % of deaths among women were attributable to smoking [20]. Insufficient physical activity accounts for 3.2 million deaths each year [20] and inadequate diets account for 11.3 million deaths and 241.4 million disability-adjusted life years [21].

Brazil has implemented several policies over the last decades that intended to reduce the prevalence of risk factors for chronic diseases. The *Action Plan for Tackling Non Communicable Diseases* (NCDs), launched by the Brazilian Ministry of Health, defines priorities for interventions and resources to combat chronic diseases and their risk factors. Furthermore, this document recognizes the importance of equity in the government's agenda, including Strategy 7, which explicitly targets monitoring of social inequalities related to risk factors, diseases, mortality, and access to comprehensive care among people with non-communicable diseases [22].

Considering the magnitude of income disparities in the country, the prevailing social differences in the national morbi-mortality rates and the impact of health related behaviors in the incidence of diseases, disabilities and early deaths, this study aimed at assessing the degree of social related inequalities in the prevalence of health behaviors of the Brazilian population by gender, educational level, self-reported skin color and possession of private health insurance in 2013.

## Methods

This is a cross-sectional population-based study, which used data from the National Health Survey (*Pesquisa Nacional de Saúde* - PNS), conducted by the Brazilian Institute of Geography and Statistics (IBGE) in partnership with the Ministry of Health. PNS is the most complete health survey ever conducted in Brazil, including questions on sociodemographic characteristics, health status, health behaviors and healthcare utilization, among others. The PNS data were collected in 2013 and 2014 using a probability sample taken in three stages. In the first stage, the primary sampling units (PSU) were composed of one or more census tracts and were selected by means of simple random sampling. In the second stage, 10 to 14 households were selected from each PSU. Finally, in the third stage, one person aged 18 years or over was selected from each household.

Interviews were pre-scheduled and data recorded on handheld computers (Personal Digital Assistants-PDAs). The PNS used three questionnaires: one referring to household characteristics, another with information about all residents, and the third soliciting information from the selected individual.

From the 64,348 households covered by the PNS, 60,202 people aged 18 years or more were interviewed, with losses totaling 20.8 % and a non-response rate of 8.1 %. Information from 49,025 individuals aged 18-59 were analyzed in this study.

Dependent variables were: current smoking (yes, no); heavy episodic drinking - HED (consumption of four or more alcoholic drinks for women and five or more for men in a single occasion during the last 30 days); being inactive in leisure time (with active individuals defined as those who practice at least 150 minutes of mild/moderate or 75 minutes of vigorous physical activity a week and insufficiently active those who practice physical activity but do not reach 150 weekly minutes); sedentary lifestyle (5 or more hours per day watching TV); low consumption of vegetables, greens and fruits (<5 times a week); ingestion of meat with excess fat (yes, no); ingestion of whole milk (yes, no). An unhealthy behavior index was developed by adding up the following points, based on the type, frequency and degree of unhealthy behaviors: 2 points for current smoking; 2 for HED; 1 or 2 points for insufficient physical activity or physical inactivity, respectively; 1 for watching TV for 5 hours or more; 1 or 2 points for consumption of vegetables, greens and fruits 2 to 4 times a week or less than twice a week, respectively; 1 point each for consumption of meat with excess fat and whole milk. The total score ranged from 0 (best) to 11 (worst) and, based on its distribution, was dichotomized into low (<7 points) or high (7 or more). Smoking and HED received 2 points each in order to avoid more under-representation of these behaviors in the combined score.

The independent variables were: gender (male and female); schooling level (college education complete, high school complete or college incomplete, elementary education complete or high school incomplete, illiterate or elementary education incomplete); skin color: white and non-white (including brown or black); and possession of private health insurance (yes, no).

Data analysis included calculation of unadjusted prevalence ratios (PR) and 95 % confidence intervals (CI) for independent variables of each health-related behaviors. The differences were tested using the chi-square test. Sex and age adjusted prevalence ratios were estimated using multiple Poisson regression. Analyses were performed using the statistical software STATA 14.0 and accounted for the sampling complex design and unequal probabilities of selection.

The National Commission of Ethics in Research (CONEP) approved the PNS project on June 26, 2013 (Regulation number 328.159). All respondents have agreed to take part in the research and signed a free and informed consent form.

## Results

We analyzed the data of 49,025 individuals of which 47.9 % (95 % CI: 47.1-48.7) were male. The average age of the population was 37 years (95 % CI: 36.8-37.2). A total of 31.9 % of the population had incomplete elementary school or no education, while 13.4 % had completed college. The individuals who declared themselves brown and black totaled 53.3 %; 70.1 % of the respondents did not have private health insurance (Table 1).

**Table 1:** Sample characteristics of Brazilian adult population (18 to 59 years of age). PNS 2013

	Total	
Variables	Number	Percent
Gender		
Male	21,365	47.9
Female	27,660	52.1
Total	49,025	100.0
Age		
18 to 29	14,321	31.8
30 to 39	14,269	26.4
40 to 49	11,405	22.0
50 to 59	9,030	19.8
Skin color		
White	18,792	46.7
Non-white (Black or brown)	29,442	53.3
Schooling		
College education complete	6,672	13.4
High school complete or college incomplete	17,739	37.4
Elementary education complete or high school incomplete	8,269	17.2
Illiterate or elementary education incomplete	16,345	31.9
Health insurance		
With private health insurance	13,025	29.9
Without private health insurance	36,000	70.1
Health behavior		
Current smoking	7,325	15.2
Heavy episodic drinking	7,629	15.7
Inactive during leisure time	33,982	67,5
Sedentary lifestyle	6,395	12.6
Consumption of fruits, vegetables and greens less than 5 times a week	38,337	76.7
Consumption of meat with excess fat	17,963	39.1
Consumption of whole milk	30,314	61.1
Unhealthy behaviors score		
0 to 1	3,436	7.6
2 to 3	12,453	26.1
4 to 6	26,811	53.3
7 or more	6,325	13.0

Analyzing health-related behaviors according to gender (Table 2), it can be seen that men had higher reporting of current smoking (*PR* = 1.70), heavy episodic drinking (*PR* = 3.01), low consumption (<5 times a week) of fruits, greens and vegetables (*PR* = 1.12), and high consumption of meat with excess fat (*PR* = 1.63) and of whole milk (*PR* = 1.03) when compared to women. Women had higher prevalence of being physically inactive in leisure time (*PR* = 0.85) and having a sedentary lifestyle (*PR* = 0.79) than men. The prevalence of having an unhealthy behaviors score of seven or more was higher among men versus women (*PR* = 2.23).

**Table 2:** Prevalence and prevalence ratios of unhealthy behaviors according to gender in the adult Brazilian population (18 to 59 years of age). PNS 2013

Health Behaviors	Prevalence (%)		PR* adjusted by age
	Female *n* = 27,660	Male *n* = 21,365	
Smoking			
Current smoking	11.4	19.3	1.70 (1.57-1.83)
Alcoholic beverage consumption			
Heavy episodic drinking	7.9	24.3	3.01 (2.83-3.33)
Physical activity/sedentarism			
Inactive during leisure time	72.9	61.6	0.85 (0.83-0.87)
Sedentary lifestyle	14.0	11.1	0.79 (0.73-0.86)
Diet			
Consumption of fruits, vegetables and greens less than 5 times a week	72.3	81.5	1.12 (1.11-1.15)
Consumption of meat with excess fat	30,0	49.0	1.63 (1.57-1.70)
Consumption of whole milk	60,3	61.9	1.03 (1.0-1.05)
Unhealthy behaviors score			
7 or more	8.2	18.2	2.23 (2.04-2.43)

*Category of reference: female

Table 3 shows behavioral prevalence rates by educational level. Respondents with less education had higher prevalence of current smoking, leisure time physical inactivity, sedentary lifestyle, and consumption of meat with excess fat and whole milk than those with more formal education. Also, those with less education had lower consumption of greens, vegetables and fruits and higher than those with more education. The prevalence of the unhealthy behavior score of seven or more increased with lower educational levels; the PR (3.74) was significantly higher among those illiterate or with incomplete elementary education as compared to those who had completed college.

**Table 3:** Prevalence and prevalence ratios of unhealthy behaviors according to schooling in the adult Brazilian population (18 to 59 years of age). PNS 2013

Health behaviors	Prevalence (%)				PR* adjusted by gender and age		
	(1)	(2)	(3)	(4)	(2)	(3)	(4)
Smoking							
Current smoking	8.4	10.3	17.1	22.8	1.30(1.10-1.56)	2.14 (1.81-2.55)	2.59 (2.21-3.03)
Alcoholic beverage consumption							
Heavy episodic drinking	15.7	15.9	16.9	14.9	0.93 (0.81-1.01)	0.99(0.81-1.02)	1.00 (0.89-1.13)
Physical activity/sedentarism							
Inactive during leisure time	49.0	61.9	68.5	81.4	1.30 (1.23-1.36)	1.43 (1.36-1.50)	1.63 (1.55-1.70)
Sedentary lifestyle	8.1	11.7	14.0	14.6	1.37 (1.15-1.62)	1.66 (1.38-2.02)	1.85 (1.54-2.20)
Diet							
Consumption of fruits, vegetables and greens less than 5 times a week	61.3	74.8	80.2	83.4	1.19 (1.14-1.23)	1.27 (1.22-1.33)	1.37 (1.32-1.42)
Consumption of meat with excess fat	28.3	36.3	43.8	44.5	1.25 (1.15-1.36)	1.51 (1.39-1.65)	1.59 (1.47-1.75)
Consumption of whole milk	49.3	63.6	64.6	61.2	1.27 (1.20-1.34)	1.29 (1.22-1.37)	1.25 (1.20-1.34)
Unhealthy behaviors score							
7 or more	5.2	9.5	14.9	19.3	1.82 (1.49-2.46)	2.86 (2.31-3.56)	3.74 (3.10-4.51)

(1) College education complete

(2) High school complete or college incomplete

(3) Elementary education complete or high school incomplete

(4) Illiterate or elementary education incomplete

*Category of reference: college education complete

Table 4 shows that individuals who declared themselves non-white (black or brown) had higher prevalence of current smoking (*PR* = 1.25), heavy episodic drinking (*PR* = 1.11), inactivity in leisure time (*PR* = 1.09), sedentary lifestyle (*PR* = 1.27), and low consumption of vegetables and fruits (*RP* = 1.15) than white individuals. The prevalence of the unhealthy behavior score of seven or more was 39 % higher in the non-white (vs. white) population.

**Table 4:** Prevalence and prevalence ratios of unhealthy behaviors according to skin color in the adult Brazilian population (18 to 59 years of age). PNS 2013

Health Behaviors	Prevalence (%)		PR* adjusted by gender and age
	White	Non-white	
Smoking			
Current smoking	13.6	16.5	1.25 (1.15-1.35)
Alcoholic beverage consumption			
Heavy episodic drinking	14.7	16.7	1.11 (1.04-1.20)
Physical activity/sedentarism			
Inactive during leisure time	65,0	69,9	1.09 (1.06-1.11)
Sedentary lifestyle	10.9	14.0	1.27 (1.16-1.40)
Diet			
Consumption of fruits, vegetables and greens less than 5 times a week	70.7	81.9	1.15 (1.13-1.17)
Consumption of meat with excess fat	38.2	40.1	1.05 (0.99-1.09)
Consumption of whole milk	60,1	62.1	1.03 (1.00-1.06)
Unhealthy behaviors score			
7 or more	10.8	14.9	1.39 (1.27-1.52)

*Category of reference: white

Table 5 shows prevalence rates for people with and without private health insurance. Among those without private health insurance there were higher prevalence rates of current smoking (*PR* = 1.73), leisure time inactivity (*PR* = 1.33), sedentary lifestyle (*PR* = 1.49), low consumption of greens, vegetables and fruits (*RP* = 1.23), and high consumption of meat with excess fat (*PR* = 1.29) and whole milk (*PR* = 1.12) than those with private insurance. The unhealthy behavior score was 78 % higher in the group without private health insurance (vs. the group with private insurance).

**Table 5:** Prevalence and prevalence ratios of unhealthy behaviors according to private health insurance in the adult Brazilian population (18 to 59 years of age). PNS 2013

	Prevalence (%)		PR* adjusted by gender and age
Health Behaviors	With private health insurance	Without private health insurance	
Smoking			
Current smoking	10.3	17.3	1.73 (1.56-1.91)
Alcoholic beverage consumption			
Heavy episodic drinking	15.9	15.6	0.95 (0.88-1.03)
Physical activity/sedentarism			
Inactive during leisure time	55.4	72.7	1.33 (1.29-1.37)
Sedentary lifestyle	9.3	13.9	1.49 (1.35-1.65)
Diet			
Consumption of fruits, vegetables and greens less than 5 times a week	65.8	81.3	1.23 (1.20-1.26)
Consumption of meat with excess fat	32.5	42.0	1.29 (1.22-1.35)
Consumption of whole milk	56.3	63.1	1.12 (1.08-1.15)
Unhealthy behaviors score			
7 or more	8.1	15.1	1.78 (1.65-1.93)

*Category of reference: with private health insurance

## Discussion

The results of this study reveal significant social inequalities in the prevalence of health behaviors in Brazil. These inequalities were expressed by differentials in the prevalence of harmful behaviors by gender, educational level, race/skin color and possession of private health insurance, with the magnitude of the inequality differing by type of behavior and stratifying variable.

Smoking prevalence was higher among males, among those with lower educational levels, in non-white individuals and in the population without private health insurance. This study identified a 15.2 % smoking prevalence: 19.3 % among men and 11.4 % in women. The Global Adult Tobacco Survey (GATS) revealed that the use of any tobacco product ranged from 21.6 % in Brazil, to 60.2 % in Russia, in the male population, and from 1.4 % in Vietnam to 42.4 % in Poland among women [23]. Globally, smoking prevalence is 4.4 times higher in men. Lower gender disparities are reported in the regions of the Americas and Europe [23] and higher inequality is reported in China, India, Japan and in the Philippines [24].

Higher smoking prevalence tends to occur among populations from lower socioeconomic backgrounds [25]. Our results are consistent with research conducted in Brazil and other countries, revealing socioeconomic inequalities based on schooling and race: smoking prevalence rates among individuals with low schooling [26, 27] and non-whites [28] were more than twice that of their more educated and white counterparts. Krieger et al. (2013), in the United States, also found a worse situation regarding health behaviors in the black population, detecting 27 % higher prevalence of smoking among blacks (versus whites), which was similar to our results (*PR* = 1.25) [28].

Smoking prevalence has declined considerably in Brazil over the past 20 years. This decline was observed across several sociodemographic groups and in different regions of the country. For instance, smoking prevalence among people aged 18 or more declined from 34.8 % in 1989 [29] to 18.1 % in 2008 [30]. Prevalence rates are still lower in the capitals of Brazilian states [27], totaling 11 % of the population aged 18-64 years. However, inequalities persist indicating the need to improve health services to support smoking cessation [31], especially for the population with low educational levels, precisely those assisted by the public health system.

Alcohol consumption is another behavior highly related to health. Harmful alcohol consumption is associated with a wide spectrum of diseases, mental disorders, and injuries, as well as social, economic and legal problems [32]. However, due to industry pressure and broad social acceptance, initiatives to control the commercialization and advertisement of alcoholic beverages are much more limited than those for tobacco. Today, in many countries including Brazil, alcoholic drinks are widely available, and they are the subject of broad advertising. These aspects are important when considering the need to reduce the unequal social distribution of the consequences of alcohol consumption.

In this study HED reached 15.7 % of the Brazilian adult population aged 18 to 59, with prevalence of 24.3 % among men and 7.9 % among women. These prevalence rates were somewhat lower than those obtained by the telephone surveys (VIGITEL) conducted with residents in Brazilian capitals aged 18 or more, (16.4 % prevalence of HED in 2013) [16].

Studies from Brazil and other countries found an increase in the rate of habitual and hazardous alcohol consumption in the last decade [31, 32], with a more dramatic increase in women [33]. Although alcohol consumption is increasing among females, the results of this study show that HED is 3 times higher in males, a finding that is consistent with the literature [32, 33]. Although consumption continues to be higher among males than females, there is evidence indicating greater vulnerability of women to the harmful use of alcohol [32].

In this study, no differences were found in the prevalence of heavy episodic drinking by educational level or private health insurance. Other studies have detected a higher frequency of habitual alcohol ingestion in the group with high socioeconomic status (assessed by different variables) [32, 33]. As to HED, somewhat discrepant results have been found, although most studies [16, 20, 34] indicate higher prevalence of HED in the segments of higher socioeconomic status. Higher hazardous consumption in the black population, as found in this study, has been reported in some studies, and has been in part attributed to racial segregation contexts [35, 36].

In this research, findings regarding physical activity point to high rates of inactivity: 67.5 % of the Brazilian adult population is inactive in their leisure time. In the population aged 18 years or more and resident in Brazilian capitals, the prevalence of physical inactivity in leisure time was 64.7 % in 2014 [22] and there is a decrease trend in this non-practice, especially among young people and those with more education [37]. In the United States, according to NHIS data from 2012, 30 % of the adult population is considered inactive and 20 % insufficiently active [38]. Brazilian women have higher prevalence of inactivity or insufficient physical activity during leisure time than men, which is consistent with the literature [22, 39].

There was significant educational inequality in the practice of leisure-time physical activity in Brazil. Respondents with less education are 63 % more likely to be inactive in leisure time than those with more education. These findings are consistent with those observed in other countries [22, 40, 41]. The inequality between brown/black and white individuals was small with rates slightly lower for non-whites. Research conducted in the USA also found lower rates of leisure-time physical inactivity among black and other minority groups. However, in one study the difference became less evident after adjustments for social class [42, 43]. Individuals without health insurance (users of the public health system) also had a higher prevalence of leisure-time physical inactivity when compared with those who have private insurance, stressing the need for maintenance of strategies to raise physical activity levels among users of public health systems.

The time spent each day watching TV, used as an indicator of sedentarism, has been investigated in relation to its negative effect on health [44, 45]. In this study, women showed higher prevalence than men of watching TV for 5 hours or more, which differs from the results obtained for adult residents in Brazilian capitals, where no difference was found between genders in 2013 or in 2014, for 3 hours or more of TV time [27, 46].

Sedentary lifestyle (watching TV 5 hours or more a day) proved to be socially inequitable. Higher prevalence rates were found in the lower educational stratum (86 % higher), in the non-white population (27 % higher) and among individuals without private health insurance (49 % higher) [37]. Mielke et al. studying the population aged 18 years and over, in a city in southern Brazil, and Clark et al (2014), with seniors in Australia, also found higher TV time in individuals with less education [37, 47].

To reduce inequalities, such as the ones identified in our study, Brazil needs to maintain and strengthen the existing policies aimed at fostering healthy behaviors in the context of primary care [48]. One of the actions of the plan for tackling chronic diseases in Brazil is the Health Academy program, which are public spaces for the practice of leisure physical activity and healthy lifestyles [22, 48].

Gender inequalities were found in the behaviors related to food. Lower prevalence of consumption of leaf vegetables, raw vegetables, and fruits was observed in men. A similar profile was detected in 2014 in the 26 Brazilian capitals and federal district [27]. A study conducted in the United States also reported lower consumption of fruits and vegetables among men [49], as well as in a research conducted in the United Kingdom, which found an average daily consumption of fruits and vegetables of 581g for women and 472g for men [50]. Moreover, a higher prevalence of consumption of meat with higher content of fat and whole milk was observed among men. A study conducted in Germany showed a higher consumption of animal fats among men, with average daily consumption of 19g compared to 13g for women [51]. A meta-analysis published in 2011 reported that the consumption of low fat milk was associated with a 16 % reduction in the risk of increasing blood pressure [52].

Our results revealed significant social inequalities in the quality of the food consumed by different groups of the population. Insufficient consumption of leaf vegetables, raw vegetables and fruits were significantly higher in the strata of lower education, in the non-white population, and among those without health insurance. These findings are consistent with other studies conducted in Brazil [46, 53, 54] and other countries. A study conducted in Australia identified higher consumption of fibers among adults with a better educational level [55]. Also, a systematic review by Darmon and Drewnowski documented an association between higher socioeconomic status and higher consumption of whole grains in most of the reviewed studies. Socially advantaged groups consumed not only larger amount of fruits and vegetables but also a greater variety of these foods [56]. A study conducted in five European countries found higher prevalence of obesity-related behaviors in neighborhoods of lower socioeconomic status [57]. As for those with health insurance, results from another Brazilian study corroborated the findings from our study regarding better food profile among individuals who had private health plan [18].

The consumption of meat with higher fat content was higher among people with lower education levels, in the non-white population, and in the stratum without a health plan. Vigitel data confirms these findings among men, but not among women [18]. However, studies conducted in Denmark, United States, Netherlands, and France observed increased consumption of lean meats, in the groups of high socioeconomic status [58–61].

Higher prevalence of consumption of milk with full-fat content was observed in the stratum of low education levels, in black and brown populations, and in individuals without a health insurance. A study conducted in the United States determined that the chance of buying low-fat milk was 50 % to 58 % lower among Latinos when compared to white communities and that the availability of skim milk or with 1 % fat milk was lower in low-income communities [62].

In Brazil, many efforts have been made to improve the quality of the Brazilian diet. Among them, we should point out the review of the 2014 Brazilian Food Guide. This innovative guide categorizes food according to the degree of processing and emphasizes the importance of homemade meals and food-based *in natura*. In addition, the new Brazilian Food Guide supports policies and programs aimed at promoting health, food safety and nutrition [63]. It should also be said that the Brazilian government regulated food labels and signed agreements with the food industry to reduce the amount of *trans* fats and sodium in processed foods [22].

We need to consider some limitations of this study. Data on health behavior are self-reported; social desirability of response and recall bias could lead to underestimation of some unhealthy behaviors. Specifically regarding the consumption of alcohol, the PNS questionnaire only asks the number of drinks and does not take into account the amount of alcohol contained in each type of alcoholic beverage. Information on the beverage consumed would increase the accuracy of the amount of alcohol consumed. However, the PNS questionnaire is similar to that of Vigitel, the Brazilian telephone survey, and several studies using these indicators confirm their validity [63–65].

On the other hand, the study has advantages. Firstly, the survey is a population-based study representative of the non-institutionalized Brazilian population. The information generated in this study is important for its focus on social inequalities in health and in health behaviors, which is scarce in developing countries and in Latin America, especially considering associations with educational level and possession of private health insurance. The study also provides important information about health behaviors in the adult population that is critical for the control of non-transmissible chronic diseases.

The results of this study revealed that a higher frequency of harmful behaviors occurs in segments with low socioeconomic background and in the non-white population, which are groups that largely depend on the Brazilian public health system for their therapeutic and preventive health care needs. These findings emphasize the role of the public health system in promoting interventions to stimulate healthy behaviors among the less advantaged groups and consequently contribute to reducing social inequalities in health. The system needs to increase the investments in health promotion, besides providing adequate health care services, including qualified and accessible support for those who intend to control their tobacco and alcohol dependence and those who need to manage their excess weight. Several government initiatives can help to reduce those inequalities, including regulation of food industries and advertisement on food and alcoholic beverages, interventions to improve the quality of school meals, the creation of public spaces to encourage the practice of physical activities among others. The concentration of harmful behaviors in some groups of people points to the need for a comprehensive approach to tackle these issues.

## Conclusion

Significant social inequalities in the Brazilian adult population, identified in this study, have profound implications for the country's morbimortality scenario in the near future. The decline that has been observed for many harmful behaviors, particularly the decline in smoking, while at the same time reducing the overall burden of early death and disabilities, can, however, be accompanied by persistent or even greater social inequalities in the incidence of these events. In general, the most socially vulnerable segments of the population have greater difficulty in adopting healthy practices. The monitoring of social disparities in terms of risk factors for NCDs, thus becomes crucial especially in Brazil, a society that continues to have one of the highest indices of income inequalities.

## Abbreviations

CI: Confidence interval
CONEP: National Commission of Ethics in Research
CSDH: Commission on the Social Determinants of Health
DALY: Disability-adjusted life year
GATS: Global Adults Tobacco Survey
HED: Heavy episodic drinking
IBGE: Brazilian Institute of Geography and Statistics
NCD: Non communicable disease
NHIS: National Health Interview Survey
PDA: Personal digital assistance
PNS: National Health Survey
PR: Prevalence ratio
PSU: Primary sample unit
SUS: Public health system
VIGITEL: Telephone-based surveillance of risk and protective factors for chronic diseases
WHO: World Health Organization
